# Qualitative Experience with an Adherence Promotion Intervention Among Individuals with Bipolar Disorder: What Is Helpful and Unhelpful?

**DOI:** 10.3390/medicina62061166

**Published:** 2026-06-16

**Authors:** Erika L. Kelley, Martha Sajatovic, Amulya Mallu, Feyi Sayo Rufai, Celeste Weise, Jessica Black, Jennifer B. Levin

**Affiliations:** 1 Department of Obstetrics and Gynecology, University Hospitals Cleveland Medical Center, 11100 Euclid Avenue, Cleveland, OH 44106, USA; 2 Department of Reproductive Biology, Case Western Reserve University School of Medicine, 11100 Euclid Avenue, Cleveland, OH 44106, USA; 3 Department of Psychiatry, University Hospitals Cleveland Medical Center, 11100 Euclid Avenue, Cleveland, OH 44106, USA; martha.sajatovic@uhhospitals.org (M.S.);; 4 Department of Psychiatry, Case Western Reserve University School of Medicine, 10524 Euclid Avenue, Cleveland, OH 44106, USA; 5 Department of Medicine, Case Western Reserve University School of Medicine, 10900 Euclid Avenue, Cleveland, OH 44106, USA

**Keywords:** bipolar disorder, medication adherence, patient perspective, qualitative research

## Abstract

*Background and Objectives*: Pharmacotherapy is a first-line treatment for bipolar disorder (BD), although half of individuals report suboptimal medication adherence. Medication adherence enhancement programs that are brief, person-specific, and remotely delivered may be an effective adjunct to treatment. The aim of this study was to qualitatively assess what individuals with BD who participated in a Customized Adherence Enhancement (CAE) program found most helpful and unhelpful about this program, focusing on elements most generalizable to medication adherence promotion. *Materials and Methods*: *n* = 14 participants with BD from the intervention arm of a randomized effectiveness trial of CAE vs. enhanced treatment–as-usual participated in one-on-one virtual, semi-structured interviews. Interviews were recorded, transcribed, and summarized using thematic content analysis to identify themes reflecting what was helpful and unhelpful about the program. *Results*: Participants identified six main themes reflecting impactful aspects of the medication adherence promotion program: practical tools (e.g., worksheets), self-awareness (e.g., identification of triggers), psychoeducation/content (e.g., specific CAE modules), emotional distress (e.g., difficult emotions arose during sessions), format (e.g., pros/cons of virtual format), and interventionist factors (e.g., rapport). *Conclusions*: Results provide participant-identified useful aspects of a remotely delivered, adjunctive medication adherence promotion program for individuals in treatment for BD in public-sector settings. Strategies for improvement and scale-up of the program include ensuring sufficient technologic and emotional support throughout the program. Future studies may investigate the longer-term impact of such interventions with multiple stakeholder input and consideration of diverse populations, settings, and cultural contexts.

## 1. Introduction

Bipolar disorder (BD) is a severe mental illness characterized by alternating episodes of depression and either mania (bipolar I) or hypomania (bipolar II). The prevalence of BD is approximately 4.4% in the United States [1]. First-line treatments include pharmacotherapy, consisting of long-term use of mood stabilizers and atypical antipsychotics [2]. About half of individuals with BD experience suboptimal adherence with medications [3,4]. Treatment often includes complex regimens, a known risk factor for suboptimal adherence [5]. Suboptimal adherence to BD medications is associated with increased risk of symptom recurrence, poorer quality of life, hospitalization, suicide attempts, and worsened comorbid conditions [3,6,7]. Interventions that enhance individuals’ adherence to BD medications and are flexible, person-centered, and adaptable across settings are critical.

Interventions that include psychoeducation and patient–provider communication may target key contributing factors to medication adherence [8,9]. Moreover, given the comorbidity between BD and substance use disorders [10], motivational interviewing strategies may be important to adherence. Customized Adherence Enhancement (CAE) is a brief intervention that is BD-specific and individually tailored to address person-specific adherence barriers using a flexible, modular format. Results of a randomized controlled efficacy trial (RCT) comparing in-person delivered CAE with an educational control, in a sample of high-risk individuals with BD, demonstrated greater improvement in adherence among those in the CAE group [11]. Remote delivery of CAE via videoconferencing by interventionists in the community where individuals get their care, and additional medication refill reminders and health promotion text messages, may allow for increased access, engagement, and decreased burden among individuals with BD, though this innovation has yet to be specifically examined. A Type 1 hybrid effectiveness-implementation project examining CAE for use in the community/public-sector care setting is currently underway [12]. This project was informed by the integrated Promoting Action on Research Implementation in Health Services (i-PARiHS) framework, representing the dynamic interaction of various factors that influence implementation success [13,14]. I-PARiHS purports that implementation success results from facilitating an innovation (e.g., intervention) with the intended recipients in their contextual setting.

In this paper, we present patient perspectives of their experiences with CAE (implemented in a community/public-sector setting using innovative remote-delivery strategies), focusing on what they found most helpful and unhelpful about the program. This allows for understanding the impact of such an intervention beyond adherence rates alone from the perspective of the recipients of care, which is consistent with person-centered models of mental health care [15]. Previous studies have evaluated adherence interventions via quantitative measurement and demonstrated efficacy [9]. We examined perceptions of the benefit vs. burden of CAE according to individuals with BD who participated in the program; and their perception of the proposed innovation of the remotely delivered format and supportive text-messaging.

The following Research Question was explored: What did individuals with BD find most helpful and unhelpful about the adherence promotion intervention?

## 2. Materials and Methods

### 2.1. Research Design

Data in this analysis were collected from a subset of participants enrolled in a Type 1 hybrid, prospective randomized effectiveness-implementation trial of CAE vs. enhanced treatment as usual (eTAU). eTAU consisted of adherence data collection using electronic pill caps (eCAPsTM) (Information Mediary Corp. Kanata, ON, Canada) and supportive/reminder text messaging (see Levin et al. 2022 [12] for information about the study protocol). CAE is a brief, remotely delivered adjunct to standard treatment consisting of up to four treatment modules (one session for each module) depending on the individual’s personal barriers to adherence, and an additional booster session. In this CAE trial, the person-specific modules were intended to be delivered via a maximum of four weekly sessions and one booster session 4 weeks after the completion of the core sessions, for a total of five possible sessions. As an effectiveness trial, timing of sessions was flexible to accommodate participants’ and interventionists’ schedules, and participants were allowed up to 6 months to complete all sessions. Sessions were delivered via videoconference (or phone in instances when participants did not have access to videoconferencing). Both CAE and eTAU arms received monthly text message reminders to refill their medications and put them in the eCAP^TM^ pill bottle (Information Mediary Corp. Kanata, ON, Canada), and monthly general adherence promotion messages. Data in the current analysis were collected from the qualitative interviews of participants at end of treatment, which, given the flexible scheduling in the effectiveness trial, varied in relation to baseline for CAE participants. A sample size of *n* = 5 to 25 is considered appropriate for qualitative research [16]. All participants were eligible for the interview, and we recruited iteratively until we reached a point of saturation in themes (i.e., at *n* = 14; see below).

### 2.2. Participants

Participants in the trial were recruited from two local public-sector mental health centers in a Midwestern metropolitan area where they were actively receiving care, given the design of the study to examine how to best integrate CAE into the workflow of public-sector care/community mental health clinics. The inclusion criteria for the trial were as follows: 18 to 89 years of age; diagnosed with BD (Type 1 or II) of at least 2 years duration using the Structured Clinical Interview for DSM-5 Research Version [17]; treated with at least one evidence-based medication for mood stabilization for at least 6 months; reported suboptimal adherence with BD medication treatments; having scores of Brief Psychiatric Rating Scale [18] ≥36, or Young Mania Rating Scale [19] ≥8, or Montgomery Asberg Depression Rating Scale [20] ≥8; having a cellular phone to receive text messages; and able to provide informed consent and complete a psychiatric interview. Participants were excluded if they were unable/unwilling to participate in interviews; at high immediate risk for harm to self or others; and/or were part of the study’s Stakeholder Advisory Board. This study was approved by the local Institutional Review Board (IRB), and all participants provided informed consent prior to participation.

For the larger trial, 269 individuals were screened to reach the enrollment target. *N* = 79 were excluded due to non-completion of study screening procedures, not meeting study criteria, being lost to follow-up prior to randomization, or withdrawal from the study. Fidelity to the intervention was assessed throughout the trial (e.g., via review of a random selection of 25% of CAE session recordings by a trained rater).

Participants included in this analysis were from the intervention arm of the trial and attended at least one session of CAE. Participants were approached by a research assistant who conducted research (but not intervention) visits. Conclusion of interviewee recruitment occurred at the point of saturation, which was determined by agreement across all three coders (see below) that new interviews did not lead to new information or themes.

The initial trial design aimed to recruit individuals with any level of participation in the trial, though participants who were lost to follow-up or who did not complete any CAE sessions did not enroll in the interviews. There were *n* = 22 individuals initially approached for qualitative interviews, with *n* = 3 who declined, withdrew, or could not be contacted and *n* = 4 who ultimately did not complete the interview due to saturation.

### 2.3. CAE Intervention Description

The four modules in CAE are selectively delivered according to the individual’s identified personal adherence barriers and preference. Personal adherence barriers were evaluated at baseline using the Attitudes towards Mood Stabilizers Questionnaire (AMSQ) and the Rating of Medication Influences (ROMI) measures. Possible modules include (1) psychoeducation (e.g., on BD and medication treatments), (2) modified motivational enhancement therapy (to address substance abuse affecting medication adherence), (3) communication with providers (e.g., communication skills-building regarding medications with a health care provider), and (4) medication routines (i.e., identification of daily routine). Based on their baseline screening of adherence barriers, individuals were ‘assigned’ relevant modules. Individuals who were not assigned to a certain module were able to personally select that module as an additional module. This provided an opportunity for the participants to select a desired module without having to personally disclose the barrier.

All interventionists were licensed social workers with mental health experience and who received additional training by the lead co-primary investigators. In addition, a risk mitigation plan approved by the IRB was in place in the event that a participant communicated, to interventionists or any other study team members including interviewers, immediate danger or was at acute risk of harm to self or others.

### 2.4. Data Collection

Data presented in the current analysis were from one-on-one, virtual semi-structured interviews conducted at the end of treatment using guides developed prior to the start of the study. The interviews were a planned portion of the larger trial to help inform future scale-up of CAE. Interview guides were structured to align with the i-PARiHS framework and identify participants’ perspectives of their experience with adherence promotion, particularly what they thought was most helpful and unhelpful about this intervention and their perception of the remote-delivery and text messaging innovation. An example question was, “Can you tell me a little bit more about the sort of things that were in the sessions that helped you the most?” Interviewers utilized prompts to glean more information about participant perspectives when warranted (e.g., “can you tell me more about that?”). All interviews were conducted via Zoom video conferencing, or phone if Zoom was not possible, and lasted approximately 60–90 min in duration.

### 2.5. Data Analyses

The interviews were recorded and manually transcribed verbatim by two research team members (AM, FR) who did not have any direct engagement with participants. EK is a clinical psychologist with experience in mixed-method research, who did not have direct contact with the participants and oversaw the qualitative analysis. EK was not part of the original trial design, but became a research team member after the trial became active and assisted with final development of the interview guides (all amendments were IRB-approved). EK trained two research team members (AM, FR) in the thematic content analysis approach [21,22]. The interviews were conducted and coded in a simultaneous fashion so that emerging insights could be incorporated into later stages of the data generation to enhance the comprehensiveness of results [23]. Prior to coding, initial general coding categories (e.g., “Helpful parts of CAE”) were created based on the interview guide and coders’ experience and discussion. Inductive coding of transcripts was iteratively used to identify themes by grouping categories (with relevant subcategories) and identifying relationships between themes via discussion among coders. Each of the three coders coded every transcript individually. A coding dictionary was developed (using Microsoft Excel) using the initial general coding categories and reviewed after a preliminary analysis of a subsample of transcripts (*n* = 3). This coding dictionary was then refined through categorization, discussion, comparison and synthetization of each code’s properties and dimensions [24]. The coders met after coding of each set of 3 transcripts for discussion and to reach consensus, and an audit trail of changes (e.g., consolidation of subcategories) was kept in a Microsoft Word 2024 document. All coders also kept reflexive memos that were discussed in meetings to reach consensus when possible or to identify alternative interpretations of quotes. Discrepancies were resolved via discussion.

## 3. Results

### 3.1. Sociodemographic Characteristics of the Sample

The average age of participants was 39.2 (SD = 11.7) years, the majority were female (*n* = 9; 64.3%), most (*n* = 11; 78.6%) identified as White, and most (*n* = 9, 64.3%) were employed full- or part-time. About one-third of the sample (*n* = 5; 35.7%) was partnered (married or cohabitating). See Table 1 for further information on the demographic characteristics of this sample. The mean number of CAE sessions attended was *n* = 4.1 (SD = 0.7). *N* = 14 (100.0%) participants received the psychoeducation module; *n* = 14 (100.0%) the communication with providers; *n* = 13 (92.9%) the medication routines; and *n* = 11 (78.6%) the motivational enhancement therapy module. See Table 2 for a breakdown of how participants were enrolled in each module (assigned based on their baseline assessment for the trial vs. the participant selecting an additional module that had not been already assigned to them).

### 3.2. Themes Derived

A total of six themes were derived from participants’ responses. Themes, associated categories, exemplary quotes, and proposed implications are further reflected in Table 3. Within most themes, participants discussed both helpful and unhelpful aspects, and some helpful aspects were reported as the opposite of unhelpful aspects.

#### 3.2.1. Practical Tools

This theme was described in a consistently positive (i.e., helpful) manner by participants. Participants identified the benefits of concrete, practical, and easy-to-use tools that they could use during and after the adherence promotion program to enhance medication adherence. Most expressed that program worksheets were valuable. The medication charts enhanced their ability to self-monitor their medication usage, provided increased accountability to themselves, increased realization of their medication usage and related implications (e.g., effects if missed a dose), and improved goal-setting. One participant described their continued use of the worksheets in monitoring their symptoms, stating, “I keep this [change plan worksheet from the motivational interviewing therapy module] on my desk and I- it’s a once-over quick glance if I am having trouble or struggling with anything regarding my bipolar disorder. I can look at this and get a quick reminder. But, it’s incredibly helpful and productive” [Participant #3057].

Most participants identified that low-tech medication organization tools, including the eCAP^TM^ and pill organizer, were beneficial for adherence and easy to use. Having transferable skills that participants could continue to use on their own was useful, including strategies for reminders to take medication, individual coping skills, and additional resources to support their mental health. One participant described the value in identifying a personalized management plan, saying, “I was able to create a plan to… overcome the barriers and be able to put something in place that will help me for the long run so that I’m taking my medication consistently” [Participant #3063].

#### 3.2.2. Self-Awareness

The program provided increased insight and self-awareness for many participants, particularly related to the identification of triggers and warning signs (of their symptoms), and increased insight into one’s own experience with BD. These responses highlight the value of personalized psychoeducation and increased insight into one’s own symptom profile in adherence promotion. For example, one participant noted, “Just looking at, uh, triggers and early warning signs, like, I thought that was really helpful” [Participant #3025]. However, considering the Emotional Distress theme described below, self-awareness via reflection of personal experiences with symptoms may also elicit psychological distress for some individuals.

Participants highlighted how the program increased their realization of the need to take medications on time, and the need to keep consistent routine/structure for management of BD. One participant identified increased insight and development of practical skills like pill reminders and routines as particularly beneficial, stating, “… it made, makes you, uh, come to the realization what’s gonna help during your bipolar. …having a routine, um, remembering to take your pills…” [Participant #3041].

#### 3.2.3. Psychoeducation/Content

Results highlighted the valuable role of personalized psychoeducation. Most participants described that the specificity of the program (to their own individual needs; focus on BD) was helpful. One participant stated, “It was nice to have something that was specifically geared towards just one thing” [Participant #3025]. Others commented on the thoroughness and specificity of the program, such as, “It was focused. It wasn’t, uhh, nonsensical or anything. It was all focused on the diagnosis and on my routines …I don’t think I would cut anything out” [Participant #3063].

Participants highlighted that content reflecting the CAE modules was useful, such as information about different types of medications and strategies for communication with a provider. For some, exposure to new and interesting information was valuable. One person noted, “How we structured the meeting so that I was able to like learn tips about, you know, when to take my meds, when to talk to my social worker or whoever. They were very informative on just what to do exactly that came in really handy. Things that I learned that I probably forgotten from years ago came back out and came to help a lot” [Participant #3086].

Alternatively, a few participants expressed that the content was not personalized enough, stating that content did not address personal comorbid conditions (such as posttraumatic stress disorder), or there was too much focus on substance use. Three participants noted the information about BD was not new to them, though two said it was helpful reinforcement of knowledge previously acquired. One participant noted, “I felt like most of the time was spent us going over things that we did in previous sessions. And he was more focused on substance abuse. …I don’t have any issues with substance abuse. And I can see other people being paranoid about something like that when you’re persistent about substance abuse” [Participant #3018]. Thus, being able to flexibly deliver psychoeducation based not only on identification of barriers before starting the program, but also on participants’ responses during sessions may be important.

#### 3.2.4. Interventionist Factors

The impact of therapeutic rapport and alliance was emphasized, especially given societal stigma around BD. Participants appreciated when the interventionist had a gentle, non-judgmental, and non-“pushy” approach. Many reported it was helpful to feel accountable to someone else and appreciated when they felt able to talk openly with the interventionist. One participant highlighted the benefit of this sense of accountability, saying, “Yes, because I felt more accountable I knew that I was going to have to speak to him again in a week and you know fill up that pill box and be asked. And then, just doing the alarm on my phone for it -I -something I never, never had done before, so it helped because I was taking my meds more often” [Participant #3123]. The same participant highlighted the importance of an attentive approach, “… it was always ‘how are you feeling right now? Do we need to call the hotline together?’ …He was always on point with it, you know, to make sure that I was okay… It just it felt like he cared” [Participant #3123]. One participant felt their interventionist did not know them well enough, which got in the way of rapport-building, stating, “I don’t know if he was having issues with tech or like what was going on. Um, I couldn’t even tell you if we are halfway, three quarters of the way. Like, he sent it [session materials] to me one time and I was like, “Oh my God. We didn’t talk about this” [Participant #3018]. For this participant, this disconnect with the interventionist got in the way of engagement, both in CAE sessions and with materials.

Participants highlighted the importance of the program being delivered by trained professionals who appeared knowledgeable. Participants appreciated that suicidal ideation (if relevant) was handled in a sensitive manner. For example, one participant stated, “I mean I thought it [suicidal thoughts] was handled delicately. …I wasn’t made to feel ashamed or embarrassed or anything” [Participant #3025]. Participants expressed appreciation when the interventionist was encouraging and flexible in terms of scheduling and communication (e.g., switching to phone if video was not working). Communication/scheduling issues with the interventionist were unhelpful. In these circumstances, participants noted that issues such as the interventionist canceling a session, being difficult to reach, or not having availability that aligned with the participant’s own availability disrupted their engagement.

#### 3.2.5. Format

Most participants indicated that the virtual, time-limited, and one-on-one structured format was helpful. Participants often appreciated the organization of the sessions, including the timing, length, and spacing of sessions and of the program and the use of a booster session. They also appreciated the organization of the program materials within the structured approach to the program, and the one-on-one individual format. One participant said, “I think there were just enough sessions. Each session covered a certain program, whether it was when to take your meds … I think every session was the just right amount of time on both sides…” [Participant #3086].

The virtual format appeared to be acceptable, convenient, and comfortable and eliminated the need for transportation for most participants. Most (*n* = 9) participants explicitly stated that they preferred this virtual format, with one stating, “I think the virtual worked a lot better for my schedule. It was more convenient versus in person, I feel like it wouldn’t be as accessible” [Participant #3125]. However, several participants identified challenges with the virtual format of the program. Technological difficulties such as internet connection issues, the link to the virtual session not working, phone storage issues (e.g., preventing adequate use of the application), and device batteries running out were challenges that some encountered. For example, one participant experienced a temporary disruption to internet connection: “Just the WIFI was an issue there for a minute” [Participant #3025].

Some participants noted anxiety associated with use of the virtual format; one described themselves as having a “phone phobia” [Participant #3086], and another noted discomfort with being on camera (“can’t hide”) [Participant #3062]. Some participants found the virtual format to be less personal than desired or than what they would expect if it had been in-person. One described that they could not observe the nonverbal communication from the interventionist as well, and said, “Maybe being in person would make it more-I don’t know what the word is-personable…” [Participant #3041]. Finally, one participant indicated that it was difficult to go over the worksheets via teleconference. “That was the only part where I was like ‘Aw man. We’re doing worksheets over Zoom.’ Obviously, that could have been part of it too. If we were in person, it probably could have been more beneficial…” [Participant #3101].

#### 3.2.6. Emotional Distress

A few participants noted that the program brought up difficult emotions or distress with reliving past experiences with BD or co-occurring conditions. For example, one described it as, “Um, a little emotional. Uh, well maybe, like minimum, like medium emotional…” [Participant #3048].

## 4. Discussion

Interventions that enhance medication adherence in individuals with bipolar disorder (BD) are critical given the significant negative impact of suboptimal medication adherence [3,5]. This qualitative study presents patient perspectives of a time-limited, adjunctive, remotely delivered, and person-specific medication adherence enhancement program (CAE) for individuals with BD receiving care in public-sector mental health settings. Results of this study are generally consistent with previous quantitative analyses (e.g., medication adherence rates), which indicate person-specific and mixed interventions (e.g., those with psychoeducation and medication regimen modification) are most beneficial [9]. This study expands upon such work utilizing the i-PARiHS [13] framework to examine participants’ experiences with this innovative medication adherence program (CAE) [12]. The qualitative approach allowed for a more detailed account of patient experience using open-ended questions, and the nature of the trial (which allowed for less rigid scheduling of sessions) may reflect a more ‘real-world’ experience of such a program. Results of this study also identify potential limitations of such programs and opportunities for improvement to maximize intervention impact.

Results showed that, for some individuals, remote delivery of a medication adherence enhancement program is acceptable and feasible and still allows for therapeutic rapport-building. However, considering the mean age, our sample may mostly represent individuals who are more comfortable with digital or remote technology. Research indicates that about one-fourth of individuals with BD do not own cell phones [25], thus limiting their access to remote interventions. Ensuring adequate technological resources and skills among recipients of the intervention may help minimize potential disruptions (e.g., internet connectivity disruption) for those who prefer remote delivery. These results should also be considered in light of possible sample selection bias, as this sample represents individuals who opted into a trial specifically examining a remotely delivered program.

Participants also highlighted the importance of having an interventionist who was non-judgmental and allowed for flexibility in communication or scheduling. Participants identified the importance of having a sense of accountability to a trusted and knowledgeable person to follow through with their goals and plans. It appears that this sense of accountability may be transferable to accountability to oneself through monitoring medication and symptoms via applied worksheets and tools. Other research shows that individuals with BD who participated in a group-based therapeutic patient education program particularly found facets of the group format helpful (e.g. having peer support; peers served as a source of other resources and hope) [26]. Thus, for some individuals, a group or peer-based format of CAE could maximize the sense of rapport-building, connection, and accountability.

Participants highlighted the value of an approach like CAE that is person-specific and tailored to address their personal barriers to adherence, such as communication with providers, medication regimens, knowledge, and substance use. This is consistent with previous research that supports person-specific tailoring of BD intervention programs [27]. Psychoeducation about BD and personal symptom profiles was critical for most participants in our study. However, consideration of the previous knowledge base may be important in tailoring programs. For some, reiteration of previously learned information may be desired, whereas for others, repetition of information may feel unhelpfully redundant. Of note, the average time since diagnosis in this sample was over 15 years; consideration of possibly outdated information previously received may be important when providing psychoeducation. Considering patients’ sources and perception of information previously acquired may be helpful to incorporate in programs. For example, another qualitative study found that individuals with BD reported that what they read online about medications strongly affected their medication-taking behavior [28]. It is also important to note that using electronic worksheets and handouts to provide information may not be a fit for everyone. Alternative methods of information provision or tailoring content for individuals with differing learning styles could be examined in future work.

Teaching practical and low-tech skills for medication regimens, such as use of phone alarm reminders and habit stacking, appear feasible and beneficial for individuals with BD receiving care in public-sector mental health settings. Results are consistent with previous research highlighting the impact of a trusting relationship with clinical providers on adherence, and the impact of increased insight on medication adherence and reduced symptomatology [29].

The adjunctive and time-limited approach to an adherence-focused intervention like CAE may help address barriers to more intensive psychosocial therapies (e.g., ability to attend multiple sessions) or enhance outcomes in individuals receiving BD interventions that focus on other aspects of the disorder, such that medication-taking behavior is lost. Given medication is a core part of treatment of BD, adherence-specific interventions perhaps should be paired with medication prescribing. Alternatively, direct integration of adherence-focused programming within existing BD interventions may be beneficial; for example, previous research shows patient-reported benefits of a behavioral activation intervention tailored to BD symptoms, which includes medication-taking behavior [30].

Limitations of this study include the short-term follow-up period, limiting the ability to determine longer-term impact of CAE. Future research using longer-term follow-ups may identify changes in participants’ perceptions over time. The large majority of participants were individuals who completed most or all of their sessions of the CAE program. Results do not reflect the experience of those who opted not to participate or who were lost to follow-up, and who may experience different barriers to engaging in a program like CAE. Given the sample was recruited from public-sector mental health treatment settings in a Midwestern metropolitan area, results may not be generalizable to other populations. Previous research indicates significant complexity of BD pathology (including genetic and environmental factors, such as the way that latitude affects sunlight) that suggests context- and person-specific needs in management approaches [31]. Future studies of participants’ experience with CAE among more diverse populations and settings (e.g., rural areas) are likely warranted. Future research may also compare outcomes between adjunct CAE and other effective treatment approaches for BD [32].

When interpreting results, it is also important to consider the potential role of social desirability bias. On most occasions, the interview was conducted by the research assistant who conducted the participant’s other research study visits (but not intervention visits) to allow for established rapport. However, this may also have led some participants to feel pressure to minimize critical feedback about the program. Given the nature of the researcher–participant relationship, participants may also have only disclosed unhelpful aspects of the program that they felt psychologically safe to share.

In addition, given the research trial design of the study, interventionists were rated on their fidelity to the exact session content and may not have had flexibility to alter or modify content sufficiently to participants’ needs (e.g., remove redundant information) or respond as they otherwise might to emotional distress (e.g., discuss previous symptom-related trauma). Finally, given the authors’ roles on the research trial team, their interpretation of the results was possibly biased by their roles and perceptions of the CAE intervention as effective.

## 5. Conclusions

Medication adherence enhancement programs that are tailored to address person-specific barriers to medication adherence, enhance personal insight into one’s symptom profile, ensure strong therapeutic rapport with a knowledgeable interventionist, and provide education on concrete and low-tech skills and tools for medication adherence appear acceptable and helpful for some individuals with BD. However, the patient’s existing knowledge base, preference for and access to digital technology, personal learning styles, and existing coping skills for management of emotional distress may affect their experience with such a program. Moreover, it is important to note these conclusions are based on participants’ perceptions rather than on a direct assessment of intervention effectiveness.

## Figures and Tables

**Table 1:** Demographics (*n* = 14).

Variables	Mean (SD)	Range	*n* (%)
Age (years)	39.2 (11.7)	23–60	
Gender			
Female			9 (64.39%)
Male			4 (28.6%)
Other			1 (7.1%)
Race			
Black			1 (7.1%)
White			11 (78.6%)
Other			2 (14.3%)
Occupation			
Full time			5 (35.7%)
Part time			4 (28.6%)
Unemployed (expected to work)			2 (14.3%)
Unemployed (disabled)			3 (21.4%)
Marital status			
Married or cohabitating			5 (35.7%)
Single, never married			6 (42.9%)
Divorced			2 (14.3%)
Widowed			1 (7.1%)
Education (in years)	13.9 (2.0)	11–17	
Years of age at bipolar disorder onset	20.6 (9.2)	6–39	
Years with bipolar disorder	18.6 (13.4)	4–54	
Insurance Type			
Medicaid			10 (71.4%)
Medicare			3 (21.4%)
Private			3 (21.4%)

Note. The percentage values associated with the “Insurance Type” variable exceed one hundred percent because participants could select more than one option.

**Table 2:** Assigned and Participant-Selected Modules (*n* = 14).

Variables	Mean (SD)	Range	*n* (%)
Assigned Modules			
Psychoeducation			13 (92.9%)
Motivational Enhancement Therapy			10 (71.4%)
Communication with Providers			13 (92.9%)
Medication Routines			11 (78.6%)
Average Number of Assigned Modules	3.4 (0.7)	2–4	
Participant-Selected Additional Modules			
Psychoeducation			1 (7.1%)
Motivational Enhancement Therapy			1 (7.1%)
Communication with Providers			1 (7.1%)
Medication Routines			2 (14.3%)
Average Number of Additional Modules	0.4 (0.6)	0–2	

**Table 3:** Description of Themes and Quotes Reflecting Individuals’ Perceptions of the Treatment Adherence Intervention, and Potential Clinical Implications.

Themes			
Theme and Categories	Example Quotes		Potential Conclusions and Implications for Enhancing Adherence in Clinical Care Settings
**PracticalTools** Worksheets, transferable skills, medication organization	“What I especially thought was amazing is the way you guys design that packet. … Where at the end, I end up with this change plan worksheet.” P #3057 (referring to change plan worksheet that is part of the motivational interviewing therapy module).	“I’m doing so much better now just like learning different tools to get me to do it. Like the alarm -I–I don’t know how I never thought to get myself an alarm. I’m taking my meds now more just because of that. So, without that, I don’t think that I would’ve gotten to the point that I’m at now. So, it was good.” P #3123	Practical, person-specific, and transferable skills can enhance personal monitoring, insight, and planning for adherence. Low-tech tools for medication regimens (e.g., pill organizers) are feasible for some individuals with BD.
**Self-awareness** Identification of triggers and warning signs, increased realization of necessity, insight	“I knew routine was good, but this almost helped me come to the realization that it is good you know? Almost having to be on the routine, …scanning the pill caps and doing the worksheet, and going to the meetings.” P #3041	“…what are personal triggers for mania, what are my personal triggers for depression. And then looking at the meds specifically and how they affect me, side effects [were helpful].” P #3057	Maximizing insight regarding personal symptoms, warning signs, triggers, and medication may support adherence.
**Psychoeducation/content** Specific CAE modules, specificity of program, previous exposure to information, personalization of the program	“I learned a lot about things that, you know, we don’t really talk about in like psychiatry and therapy sessions…” P #3125	“I think I learned a lot of stuff about bipolar disorder and I’ve got more information on it, which was helpful to me because I have known that I’ve had it for 4 years but it’s always good to kind of learn more about it…” P #3116	Incorporate person-specific information in psychoeducation. Consider patient’s previously learned knowledge (e.g., reiterate information to strengthen information previously learned, or reduce unnecessary redundancy based on patient’s personal knowledge base and preference).
	“I just feel like for me, in recovery for mental health, I want to talk to somebody. I don’t want to look at worksheets …It’s not personal. This is a very personal experience for everybody and it is very hard to put a paper in front of me.” P #3101	“…maybe some of like the more basic explanation of bipolar disorder [was less helpful]. But, I will say that just because it didn’t necessarily help me doesn’t mean it wouldn’t help other people because I have had the privilege of like going to a psychiatrist and a therapist so it’s not like I’m coming in with like zero knowledge of bipolar.” P #3116	Ensure interventionists are aware of the unique symptom profile and previous knowledge of the individual with BD. Ensure approach is aligned with goals and needs of the specific patient.
**Format** Participatory format, organization of materials, organization of sessions, individual format, convenience of virtual technology, technical challenges of virtual format	“…better because of the structure of having worksheets to do and to read over as opposed to sometimes when I’m just free flow talking. I like the structure of our meetings a lot—the question and answer type session. I thought that was really good.” P #3086	“With bipolar, some days, I don’t wanna leave the house. So, it’s [virtual format] really nice and convenient.” P #3084	A one-on-one, structured, and virtual format is accessible and feasible to some individuals with BD and may overcome symptom-based barriers to treatment (such as social withdrawal)
	“At first, it gets anxious like right before you start, for me at least. I have a phone phobia, so it’s a little hard for me to get started. But, once you get going and get used to the process, it’s not hard at all.” P #3086:	“I would have engaged with it if it would have been in person or there was a requirement for me to come in person. I like having that personal connection because of my struggle of coming back from addiction.” P #3057	Ensure there is sufficient time and resources to address training and troubleshooting in the use of telehealth technology. Consider patient experience and preference for/against remote and/or digital format.
**Interventionist factors** Therapeutic rapport, trained professionals, encouraging, accountability to others, sensitivity to suicidality, flexibility, communication/scheduling issues	“Wanting to be honest with them, and you know, being truthful, you know, was–was good to be able to be open about it… knowing I wasn’t going to be judged about.” P #3063	“it’s easier to do since there’s no opinion, I can form my own. So, there’s a lot of shame and guilt tied into when people give opinions and say you should do this because of this….I’ve been in recovery for a while, so I can pretty much decide how I feelabout certain things and feel confident about it, so that’s been easier.” P #3101	Ensure programs are conducted by trained interventionists with specific expertise in BD. Consider the impact of social stigma on therapeutic rapport.
	“…knowing that I was going to be accountable to somebody was helpful in the beginning and then, it kinda slacked off because my counselor was …in between jobs and different things or whatever. I kinda got lost in the middle so that was, that was unfortunate.” P #3063	“We both had a lot of troubles connecting like a lot of it was on his end. Sometimes it was on my end.” P #3018	Ensure interventionists are responsive and flexible to individual needs. Ensure interventionists are reliable in scheduling and maintain communication between sessions (e.g., for scheduling).
**Emotional distress** Difficult emotions, reliving experiences	“With me being so worked up and like trying to calm down from the stuff like going through it and bringing it up fresh again, that kinda was hard to relive it all over again.” P #3025		Ensure programs are conducted by trained interventionists with specific expertise in BD and management of acute distress. Consider incorporating trauma-informed care components into CAE, and/or consider teaching the patient skills for managing emotional distress during sessions.

P = Participant. Bold text indicates the name of the theme, with categories indicated below.

## Data Availability

This study dataset is entered into the common informatics platform by the U.S. National Institute of Mental Health (NIMH), National Database for Clinical Trials Related to Mental Illness (http://ndct.nimh.nih.gov, NDCT).

## Abbreviations

The following abbreviations are used in this manuscript:

BD	Bipolar disorder
CAE	Customized adherence enhancement
eTAU	enhanced treatment as usual
i-PARiHS	integrated Promoting Action on Research Implementation in Health Services
